# Immune Pathways in Atopic Dermatitis, and Definition of Biomarkers through Broad and Targeted Therapeutics

**DOI:** 10.3390/jcm4050858

**Published:** 2015-04-29

**Authors:** Yasaman Mansouri, Emma Guttman-Yassky

**Affiliations:** Department of Dermatology, Icahn School of Medicine at Mount Sinai, 5 East 98th Street, New York, NY 10029, USA; E-Mail: yamansouri@gmail.com

**Keywords:** atopic dermatitis, eczema, biomarker, translational revolution, T-cells, intrinsic, extrinsic, immune phenotype

## Abstract

Atopic dermatitis (AD) is the most common inflammatory skin disease. Recent research findings have provided an insight into the complex pathogenic mechanisms involved in this disease. Despite a rising prevalence, effective and safe therapeutics for patients with moderate-to-severe AD are still lacking. Biomarkers of lesional, nonlesional skin, and blood have been developed for baseline as well as after treatment with broad and specific treatments (*i.e.*, cyclosporine A and dupilumab). These biomarkers will help with the development of novel targeted therapeutics and assessment of disease reversal, with the promise of a more personalized treatment approach. Since AD involves more than one subtype (*i.e.*, intrinsic/extrinsic, pediatric/adult,* etc.*), these molecular fingerprints needs to be validated in all subpopulations with AD.

## 1. Introduction

Atopic dermatitis (AD) causes substantial morbidity and greatly impacts quality of life of affected individuals and their families [1]. It is the most common chronic inflammatory skin disease [2] with a prevalence of 2%–10% among adults and up to 15%–30% among children [3,4], with significant geographic variations. While around 85% of patients first develop the disease in childhood, adult-onset AD has also been recognized [5]. The incidence of AD has been rising over the last decades, especially in industrialized nations. Despite the high frequency of AD and its negative impact on both patients and their families, its exact pathogenesis remains elusive. It is, however, evident that the pathogenesis of AD is complex and involves environmental and genetic factors, barrier defects with increased trans-epidermal water loss (TEWL) and decreased lipids [6], reflecting underlying alterations in keratinocyte differentiation [5,7], immune dysregulation [5], marked epidermal hyperplasia in chronic lesional skin [7] and increased susceptibility to infections [8].

AD often starts in childhood, and can be the initial manifestation of the “atopic march”, which includes asthma, food allergy and allergic rhinitis [9]. In the past, AD was thought to be a single disease, while recent studies suggest that various phenotypes and endotypes exist [10]. AD poses a large unmet need for safe and effective therapeutic modalities. With our improved understanding of the complex pathogenic mechanisms involved in this multifactorial disease, it is becoming increasingly evident that the selection of the therapeutic agent should not be based on serendipity, but on the different subsets of the disease phenotype, its severity, and the ethnic background of the patient. Due to the clinical manifestations of AD, such as erythema and lichenification, it can be challenging to quantify its activity clinically. In addition, AD has a high placebo response rate [11,12], which is a great disadvantage when undertaking clinical trials of therapeutics. We therefore require a set of biomarkers, both for disease activity, and for successful treatment response, to allow us to accurately assess reversal of the core disease pathology.

Two main hypotheses were suggested for the primary AD pathogenesis. In the “outside-in” hypothesis, an intrinsic epidermal-barrier dysfunction is the initial insult that precedes immune activation [13,14]. The defective stratum corneum allows TEWL, allergen penetration, increased susceptibility to colonization and infection by microbial pathogens, and systemic allergen sensitization, predisposing to both food allergy and asthma [15]. The “inside-out” hypothesis suggests that the disease is primarily cytokine-driven with resultant, reactive epidermal hyperplasia caused by immune activation [16]. These alternate hypotheses have been debated for some time. The discovery of mutations in the gene encoding filaggrin (*FLG*) in AD in 2006 [17] led to a theory of *FLG* mutations as a potential cause of AD, and this temporarily shifted the hypothesis of the primary pathogenic mechanism of AD to one of epithelial barrier dysfunction [18]. However, only a subset of AD patients (up to 30% to 40%) were found to carry such a mutation, with significantly lower to even absent *FLG* mutation rates in certain ethnic groups [19,20,21]. More recent studies examining the genomic and histologic profiles of AD, in particular by comparing clinically unaffected or nonlesional (NL) to lesional AD skin (LS), and acute (defined as lesions of less than 72 h duration that appeared on previously uninvolved skin) to chronic AD lesions, and further evaluation of potential similarities and differences between AD and psoriasis, have considerably enhanced our understanding of the pathogenic mechanisms of AD. While these findings indicate that AD is primarily an immune-mediated disease, we are starting to recognize that the pathogenesis of AD may be more complex than previously implied by the above two hypotheses [22,23,24].

The progress made in the understanding of the pathogenesis of psoriasis and development of biomarkers of psoriatic disease and therapeutic response over the last decade has somewhat assisted the evolving pathogenic and therapeutic development in AD. Biomarkers can be divided into those of disease and those of therapeutic response, which do not necessarily correlate. There are several reasons why biomarker development is of particular importance in the AD cohort. In AD, the disease phenotype is visually evident and measurable at a clinical level by using several scoring methods, such as SCORing for Atopic Dermatitis (SCORAD), Eczema Area and Severity Index (EASI), Investigator’s Global Assessment (IGA), and pruritus scores,* i.e.*, visual analogue scales [25,26,27,28]. It is very difficult to quantify improvement in erythema and lichenification particularly in patients who received short-term treatment, with more subtle clinical effects, leading to high placebo rates (approximately 20%–23%) in AD [11,29,30],* vs.* only 2%–5% in psoriasis [31,32,33]. As with other inflammatory diseases, such as psoriasis [34,35,36,37,38] we are in a new era of molecular medicine where translational research accelerates therapeutic development. Pathway-targeted therapeutics are tested on the basis of a hypothesis, and to prove the hypothesis we need to demonstrate that suppression of the pathway is associated with disease improvement. Ideally, we would have a read-out of disease improvement in a shorter time frame and with greater accuracy than provided by current clinical scoring methods. We therefore need reliable biomarkers of disease activity that can be reversed by successful therapeutics, and reliable biomarkers of suppression of targeted pathways (molecules that are directly or indirectly targeted by a drug), as well as dose-response correlations that are more robust than clinical scoring provides. In fact, in AD this is even more critical than in psoriasis, since the placebo responses are much higher in AD [11,29,30,31,32,33].

## 2. New Understanding of Immune Pathways Underlying AD and Definition of Cutaneous Biomarkers of Disease

AD is characterized by excessive T-cell activation, with significant skin infiltration by T-cells and dendritic cells (DCs). Both Th2 and Th22 activation are hallmarks of AD, with some Th17 and Th1 components. An important breakthrough in the understanding of disease pathogenesis was provided by studies investigating genomic and histologic profiling of AD skin [39,40]. These studies have defined sets of biomarkers of the lesional AD skin, which are correlated with disease activity [39,40]. These include markers of epidermal hyperplasia (epidermal thickness, K16 and Ki67 staining), as well as markers of cellular infiltrates, including T-cells (CD3+ and CD8+), and several dendritic cell subsets (CD11c+, CD1a+, inflammatory dendritic epidermal cells (IDECs), as quantified by CD206+ and FceR1+ cells, OX40L+, TRAIL+, plasmacytoid/BDCA+2, mature/CD83+). The terminal differentiation markers loricrin (LOR), periplakin (PPL), and filaggrin (FLG) are inversely correlated with disease severity.

Among the immune biomarkers are markers of inflammatory mediators (MMP12), Th2 (IL-13, CCL11, CCL17, CCL18, CCL22), Th1/IFN (CXCL10), Th22 (IL-22), Th17 (IL-23p19, IL-23p40, CXCL1, PI3/elafin), and the IL-17/IL-22 induced S100A7, S100A8, S100A9, and S100A12 [40]. These cellular and molecular biomarkers represent the inflammatory skin phenotype of lesional AD skin.

The non-lesional/NL skin of AD patients (“background skin phenotype”) demonstrates distinct differences to normal skin, with an intermediate phenotype between normal and LS skin [39]. Significant increases in epidermal thickness and Ki67+ cell counts are found from normal to NL skin, with additional increases in Ki67 counts from NL to LS skin. In contrast to normal skin, which does not demonstrate any suprabasilar K16 expression, NL skin has variable K16 staining with occasional continuous suprabasilar staining. Nonlesional AD skin is also markedly abnormal with regards to epidermal differentiation and expression of some inflammatory gene products [39]. Levels of terminal differentiation genes, including LOR, FLG, CDSN, LCE proteins, psoriasis 1 candidate 2 (PSORS1C2), and SPRR4, which are significantly suppressed in LS skin, are also downregulated in NL skin compared to normal skin. Significant increases in Th2, Th22, and Th1-cytokines further characterize NL skin. Interestingly, NL skin samples cluster according to their respective SCORAD scores, possibly due to higher systemic inflammation with higher SCORAD. Expression of several immune genes in NL skin shows positive correlations with disease activity (as measured by SCORAD). These include Th2-related cytokines (CCL22, CCL18, and IL-13), the Th22-related IL-22, and S100A7, Th1-related products (MX1). Epidermal thickness, and CD3+ T-cell and CD11c+ DC counts in NL skin, were also correlated with SCORAD. Significant negative correlations with SCORAD were found in NL skin for differentiation markers. These offer another set of biomarkers specific to nonlesional AD skin. While the majority of the currently known biomarkers of AD disease activity are found in the skin, a number of serum biomarkers correlating with disease activity have also been identified. These include IgE, eosinophils, eosinophilic cationic protein (ECP), as well as the Th2 chemokines CCL17, CCL18, CCL22, CCL11, and CCL26, and the cytokines IL-13, IL-31, and IL-22 [41,42,43,44]. With the exception of IL-31, CCL17, ECP, eosinophils, and IgE, none of these serum biomarkers have been shown to also be treatment response biomarkers [45,46,47].

## 3. Classification of AD

AD can be divided into several subtypes, which differ in terms of prevalence, age of onset, clinical features, and response to treatment [6,10,15,39,48,49,50,51]. The subtypes currently best understood are the acute and chronic, and extrinsic and intrinsic subtypes [7,51,52,53,54,55,56,57,58,59,60,61,62,63,64,65]. Other phenotypes of the disease may be distinguishable in terms of ethnic origin (European* vs.* African American, Asians,* etc.*), age (pediatric* vs.* adult AD) [55], and response to treatment.

Although historically considered a biphasic disease, with a predominant Th2 response in acute disease, and a switch to a Th1 response in chronic disease [66], our recent study of paired non-lesional, acute and chronic skin lesions from AD patients demonstrated that onset of acute AD is characterized by Th2 and Th22 activation with intensification of these pathways (rather than a switch to a primarily Th1 polarization) in chronic disease, where an increase in Th1-related markers is also seen [7]. Both Th2-related (IL-4, IL-13, and IL-31) and Th22-related cytokines (IL-22) are significantly overexpressed in acute AD lesions [7]. IL-31 is also increased in the serum of AD patients [43,67], and increased blood levels of this itch-promoting cytokine positively correlate with disease severity [43,44]. The onset of acute AD lesions is also characterized by marked increases in T-cells and DCs. Some increases are also seen in Th17/IL-23-related cytokines and products (including IL-17A, CCL20, PI3/elafin, lipocalin 2 (LCN2)), and IL-23 [7]. With the development of acute AD lesions, there is marked epidermal hyperplasia, as demonstrated by the increase in epidermal thickness and proliferation markers Ki67 and K16, with further increases in chronic lesions [7]. This may be explained by the effects of the IL-22 cytokine [65]. Striking increases in acute AD are also seen in the S100A7, S100A8, and S100A9. IL-22, IL-17, or both may cause the activation of these S100 genes, as these cytokines have been shown to upregulate S100 production* in vitro* [68,69]. As progression to chronic disease occurs, further significant increases are seen in numbers of T-cells and DCs, as well as Th22/IL-22-related products (S100A7-9 and IL-32), Th2 cytokines and chemokines (IL-5, IL-13, IL-31, IL-10, CCL5, CCL13, and CCL18), and Th1-related molecules (INF-γ, MX1, IL-1β, CXCL 9-11). The magnitude of Th17 activation in chronic AD lesions is similar to acute AD, with no further intensification seen. In contrast to the other Th2 cytokines, IL-4 and its receptor appear to decrease rather than increase with disease chronicity. Nevertheless, IL-4 is amongst the disease biomarkers that correlate with disease activity in chronic AD. Other predictors of SCORAD index in chronic lesions are IL-31, INF-γ, IL-17A, and PI3 (Table 1) [7]. Among the best skin biomarkers identified in acute lesions are IL-31, IL-4, CCL18, CCL13, IL-22, S100A7-9, S110A12, and tumor necrosis factor-related apoptosis-inducing ligand [TRAIL] [7].

**Table 1:** Biomarkers of disease activity.

	Lesional Skin	Nonlesional Skin
Suárez-Fariñas *et al.* * J. Allergy Clin. Immunol.* **2011**, *127*, 954–964.	Markers of epidermal hyperplasia:	Epidermal thickness and Ki67 CD3+ T-cell and CD11c+ DC counts Th2 (CCL22, CCL18, and IL-13) Th22 (IL-22) S100A7 Th1 (MX1) *LOR*, * FLG*,* CDSN*,* LCE*; PSORS1C2 and SPRR4 (inverse correlation)
	Epidermal thickness, K16 and Ki67	
	Markers of cellular infiltrates:	
	T-cells (CD3+ and CD8+),	
	DC subsets (CD11c+, CD1a+, IDECs [FceR1, OX40L, TRAIL] plasmacytoid/BDCA+2, CD83)	
	Inflammatory mediators: MMP12	
	Th2-related products (IL-13, CCL11, CCL17, CCL18, CCL22)	
	Th1/IFN (CXCL10)	
	Th22 (IL-22)	
	Th17 (IL-23p19, IL-23p40, CXCL1, PI3/elafin)	
	S100A7, S100A8, S100A9, and S100A12	
	*LOR*,* PPL*,* FLG* (inverse correlation)	
Gittler *et al.* *J. Allergy Clin. Immunol.* **2012**, *130*, 1344–1354.	Acute lesions:	
	Epidermal thickness, Ki67, K16	
	IL-31, IL-4, CCL18, CCL13, IL-22, S100A7-9, S110A12	
	TRAIL	
	Chronic lesions:	
	Epidermal thickness, Ki67, K16	
	IL-4, IL-31, INF-γ, IL-17A, and PI3	
Suárez-Fariñas *et al.* *J. Allergy Clin. Immunol.* **2013**, *132*, 361–370.	Intrinsic AD:	
	CCL20, IFN-α, and IL-1β	
	Extrinsic AD:	
	Serum IgE levels (highly significant)	
	Th2-cytokines (IL-4, IL-5)	
	Th2-cytokines (IL-21 and IL-19)	
	*LOR* and *PPL * (inverse correlation)	

## 4. Extrinsic* versus* Intrinsic AD

Extrinsic and intrinsic AD represent approximately 80% and 20% of the adult AD patient population, respectively [51]. Patients with extrinsic disease have high levels of serum IgE, and often also a personal or family history of atopy and specific IgEs to food or aeroallergens [62], while intrinsic AD patients have normal total IgEs, absence of other atopic diseases, negative allergen-specific IgEs, and lower rates of filaggrin mutations [56,61]. Interestingly, several studies report a female preponderance in the intrinsic subgroup [57,70,71,72]. Despite these differences, both subtypes share a similar clinical pattern, with intensely pruritic, poorly defined erythematous, eczematous patches with a predilection for the skin flexures and the face. Our recent study [51] has shown important differences in T-cell polarity between these two subtypes, with possible therapeutic implications. Intrinsic disease was shown to be characterized by higher immune activation overall, with similar Th2 activation to extrinsic AD. A significantly higher Th22 (IL-22) and Th17/IL-23 (IL-17A, IL-12/IL-23p40, elafin, CCL20, S100As) activation were seen in intrinsic compared with extrinsic AD. The significant involvement of the Th2 pathway in both subtypes suggests that a Th2 bias is not the only cause of high IgE levels in extrinsic patients, but other factors must play a role in IgE activation. A number of correlations with disease activity unique to either of these subtypes were found, including CCL20, IFN-α, and IL-1β, which only correlate with disease activity in intrinsic lesions, while serum IgE levels (highly significant), the Th2-cytokines (IL-4, IL-5), as well as the Th2-promoting cytokines (IL-21 and IL-19) were associated with disease activity in extrinsic lesions only (Table 1). A negative link between differentiation markers (*LOR* and *PPL*) and the SCORAD was only seen in extrinsic AD [51]. These data may suggest that a personalized medicine approach might be possible for AD patients, with therapeutic options extending beyond targeting the Th2 pathway, to treatments targeting the Th17 and Th22 pathways, as should be clarified through future clinical trails [51].

## 5. Phenotypic Variations Based on Ethnicity

Several differences have been noted in AD phenotypes based on the ethnic background of patients. Compared to Caucasians, AD in African Americans is associated with lower rates of *FLG *mutations, higher prevalence [73,74], as well as more severe [75] and treatment-resistant disease [76,77]. Furthermore, normal skin of African Americans is characterized by a lower ceramide-to-cholesterol ratio [78] and greater TEWL [79,80]. A higher prevalence of AD has also been reported in Asian and Hispanic AD populations [81,82], and an association between central obesity and systolic hypertension and AD has recently been reported in a pediatric cohort of Hispanic and Asian heritage [83]. Despite these observed racial variations, there is a lack of data regarding possible differences in immune activation and epidermal responses in different ethnic populations. Studies of molecular and cellular biomarkers, as well as systemic inflammation are necessary to improve our understanding of different subsets, and to allow us to develop targeted therapeutics for specific AD phenotypes.

## 6. Definition of Disease Response Biomarkers

Defining biomarkers in AD does not only enable us to better understand the disease pathogenesis by providing the “molecular fingerprinting” of AD, but it also assists in the development of novel targeted therapeutics and assessment of successful disease reversal, similar to psoriasis, where biomarker data are now integrated into the early phase development of most new drugs [34].

Despite the high prevalence of AD, which has increased two- to threefold during the last century [5], and the great economic burden it poses [84,85,86], treatment options for patients with moderate-to-severe disease are very limited [34,37,87]. Therapeutic agents used in the management of AD mostly provide symptomatic relief, in the form of topical emollients and topical anti-inflammatory agents, with limited, unspecific options for moderate-to-severe disease [88]. Cyclosporine A (CsA) is a potent immunosuppressant that is highly effective in moderate-to-severe AD [89,90,91,92], and is the only systemic agent approved for this indication in some countries, although not approved for AD in the US [93]. Broad immune suppressive agents such as CsA, systemic corticosteroids, and NB-UVB phototherapy have been used for decades in more severe cases of AD [94,95], however, their selection was largely based on serendipity without a detailed understanding of their molecular effects in AD.

We have tested the hypothesis that effective clinical reversal of AD disease activity is coupled with suppression of the immune phenotype and reversal of the epidermal pathology in tissues using studies with broad agents such as NB-UVB, CsA, and the first targeted therapeutic, dupilumab, paving the way for the long-awaited targeted therapeutics (Table 2) [11,23,24,40]. The reversal of the AD disease phenotype in skin tissues consists of a composite of inhibition in inflammatory markers and reversal of the abnormal epidermal phenotype. Across NB-UVB and CsA, we identified common disease reversal biomarkers that show significant improvement following treatment. These include measures of epidermal hyperplasia (epidermal thickness, and protein and mRNA expressions of K16 and Ki67), S100 responses, T-cell (CD3+ and CD8+) and DC (*i.e.*, CD83+, CD206, FceRI+, *etc.*) cellular infiltrates, and cytokines and chemokines defining Th2-,Th22-, and Th17-activation (including IL-13, IL-22, IL-19, MMP12, S100A12, CCL17, CCL18, CCL22, elafin/PI3, IL-23A, *etc*.) with modest effects on terminal differentiation genes (e.g., *LOR* and *FLG*) [23,40].

**Table 2:** Treatment response biomarkers.

	**Cyclosporine A**
Khattri *et al.* * J. Allergy Clin. Immunol.* **2014**, *133*, 1626–1634.	Epidermal thickness, K16 and Ki67 S100 responses
	T-cell (CD3+ and CD8+)
	DC (*i.e.*, CD83+, CD206, FceRI+, *etc.*)
	MMP12, IL-23A
	Th2 (IL-13, IL-19, CCL18, CCL17, CCL22, CCL26)
	Th22 (IL-22, S100A7-9, S100A12)
	Th9 (IL-9)
	Th1/IFNγ (CXCL10)
	Th17 (Pi3/elafin, CXCL1)
	*LOR*, *FLG*, CLDN8 (inverse correlation)
	**NB-UVB**
Tintle *et al.* *J. Allergy Clin. Immunol.* **2011**, *128*, 583–593.	Epidermal thickness, K16 and Ki67 S100 responses
	T-cell (CD3+ and CD8+) and DC (*i.e.*, CD83+, CD206, FceRI+, *etc.*)
	Th2-, Th22-, Th17-related molecules (IL-13, IL-22, IL-19, MMP12, S100A12,
	CCL17, CCL18, CCL22, elafin/PI3, IL-23A)
	IL-22
	*LOR* and *FLG * (inverse correlation)
	**Dupilumab**
Beck LA *et al.* * New Engl. J. Med.* **2014**, *371*, 130–139. and Hamilton *et al.* *J. Allergy Clin. Immunol.* **2014**, *134*, 1293–1300.	S100A7, S100A8, S100A12, MMP12,
	Th2 (CCL13, CCL17, CCL18, CCL26)
	IL-17 (elafin/PI3, IL-23p19/IL23A)
	Epidermal thickness, K16, Ki67, CLDN8 CLDN11, *FLG* and *LOR*
	(inverse correlation)

IL-22 is the only cytokine significantly correlated with the reduction in SCORAD scores following NB-UVB therapy, and it also appeared to be the most highly correlated amongst several disease characteristics. Compared with NB-UVB, CsA therapy results in more rapid and more significant improvement in the genomic and cellular phenotypes, including larger effects on non-lesional skin [23]. Greater reductions are seen in Th2, Th22, Th9, and some Th1- and Th17-related markers. CsA leads to histological resolution of epidermal hyperplasia, reversal of abnormalities in epidermal thickness, expression of K16 and Ki67, and up-regulation of *LOR* and *FLG*. CsA, a non-specific immune antagonist, significantly suppresses all immune axes involved in AD, in particular Th2-related cytokines and chemokines (IL-13, IL-19, CCL18, CCL17, CCL22, CCL26), Th22- (IL-22, S100A7-9, S100A12), and Th9- (IL-9) related products with some reduction in Th1/IFNγ (CXCL10) and Th17 (Pi3/elafin, CXCL1) markers, and significant up-regulation of terminal differentiation genes, such as claudin 8 [23].

Dupilumab, a fully human monoclonal antibody targeting the IL-4 receptor-α, blocking both IL-4 and IL-13 signaling, is a newly developed agent, and the first targeted treatment to show successful results in early clinical trials for AD [11,24]. Dupilumab has significant dose-dependent clinical efficacy in AD, irrespective of serum IgE levels [11,24], and it also potently reverses the molecular fingerprint of AD. It shows significant dose-dependent down-regulation of S100A7, S100A8, S100A12, and MMP12, as well as impressive inhibition of Th2-related chemokines (CCL13, CCL17, CCL18, CCL26) after only four weeks of treatment, and reverses the epidermal phenotype (epidermal thickness, K16, MKi67) without major effects on the Th1 axis. While there is no marked inhibition of IL-17A or IL-22 with this IL-4 receptor-α antagonist, there is a marked decrease in IL-17-related products (elafin/PI3, IL-23p19/IL23A, and S100A8), with parallel increases in barrier genes (CLDN8 and CLDN11) [24], and a trend towards increases in expression of differentiation genes such as *FLG* and *LOR*. Interestingly, an overall exacerbation of the inflammatory lesional AD phenotype was observed in the placebo-treated cohort, despite “clinical improvements” seen in these patients, further confirming the importance of biomarker studies in AD [24]. These data establish IL-4/IL-13 as major pathogenic cytokines in AD that drive a complex Th2-centered inflammatory axis. More comprehensive and long-term studies are under way to evaluate long-term disease suppression with dupilumab.

Led by the observation that AD lesions tend to recur in the same sites following successful treatment, the residual expression of disease-related genes, named “residual disease genomic profile” (RDGP) of patients has also been assessed [49,50]. The RDGP includes sets of genes that do not improve despite clinical resolution of AD lesions, and are likely to predispose to disease recurrence [49]. The RDGP in clinically resolved skin lesions after NB-UVB treatment includes immune egenes (e.g., MX1, S100A7, S100A8, IL-8, CXCL1, CXCL2), and structural genes (aquaglyceroporin 7/AQP7 (a marker of epidermal hydration, CLDN8, CLDN23, peroxisome proliferator-activated receptor γ/PPARG and leptin/LEP) [49]. Although the RDGP after CsA treatment is more modest than after NB-UVB, key AD genes are part of it (e.g., IL-31, *etc.*), possibly explaining the earlier disease relapse after cessation of CsA treatment [49,50].

## 7. Conclusions

We are at the beginning of an exciting era of increased understanding of pathogenic AD circuits, leading to a translational revolution in this disease [34]. By defining disease and treatment response biomarkers, and comparing different AD subtypes, we are slowly advancing towards the possibility of a personalized medicine approach in AD [96]. For future development of targeted therapeutics, it will be prudent to incorporate comprehensive biomarker studies, with the aim to also target both the RDGP and the background disease phenotype, and to allow us to predict whether potential new therapies are likely to be efficacious and have the potential to reverse pathologic hyperplasia. This approach will allow us to not only treat active disease, but to also avoid recurrences of AD, and to potentially even halt the atopic march.
